# The Role of Transforming Growth Factor β in Cell-to-Cell Contact-Mediated Epstein-Barr Virus Transmission

**DOI:** 10.3389/fmicb.2018.00984

**Published:** 2018-05-15

**Authors:** Asuka Nanbo, Makoto Ohashi, Hironori Yoshiyama, Yusuke Ohba

**Affiliations:** ^1^Department of Cell Physiology, Faculty and Graduate School of Medicine, Hokkaido University, Sapporo, Japan; ^2^Department of Oncology, University of Wisconsin, Madison, WI, United States; ^3^Department of Microbiology, Shimane University Faculty of Medicine, Izumo, Japan

**Keywords:** Epstein-Barr virus, cell-to-cell contact-mediated transmission, viral replication, TGF-β, exosomes

## Abstract

Infection of Epstein–Barr virus (EBV), a ubiquitous human gamma herpesvirus, is closely linked to various lymphoid and epithelial malignancies. Previous studies demonstrated that the efficiency of EBV infection in epithelial cells is significantly enhanced by coculturing them with latently infected B cells relative to cell-free infection, suggesting that cell-to-cell contact-mediated viral transmission is the dominant mode of infection by EBV in epithelial cells. However, a detailed mechanism underlying this process has not been fully understood. In the present study, we assessed the role of transforming growth factor β (TGF-β), which is known to induce EBV's lytic cycle by upregulation of EBV's latent-lytic switch *BZLF1* gene. We have found that 5 days of cocultivation facilitated cell-to-cell contact-mediated EBV transmission. Replication of EBV was induced in cocultured B cells both with and without a direct cell contact in a time-dependent manner. Treatment of a blocking antibody for TGF-β suppressed both induction of the lytic cycle in cocultured B cells and subsequent viral transmission. Cocultivation with epithelial cells facilitated expression of TGF-β receptors in B cells and increased their susceptibility to TGF-β. Finally, we confirmed the spontaneous secretion of TGF-β from epithelial cells, which was not affected by cell-contact. In contrast, the extracellular microvesicles, exosomes derived from cocultured cells partly contributed to cell-to-cell contact-mediated viral transmission. Taken together, our findings support a role for TGF-β derived from epithelial cells in efficient viral transmission, which fosters induction of the viral lytic cycle in the donor B cells.

## Introduction

Epstein–Barr virus (EBV), a ubiquitous human gamma herpesvirus, infects approximately 95% of the population worldwide and establishes persistent lifelong, mostly asymptomatic infection. EBV infection is associated with various lymphoid and epithelial malignancies such as Burkitt's lymphoma (BL), Hodgkin's disease, gastric carcinoma (GC), and nasopharyngeal carcinoma (NPC) (Longnecker and Cohen, 2013).

Previous studies demonstrate that the efficiency of EBV infection in epithelial cells is significantly up-regulated by coculturing them with B cells latently infected by EBV relative to infection with cell-free virus (Imai et al., 1998; Chang et al., 1999; Speck and Longnecker, 2000; Nanbo and Takada, 2002; Shannon-Lowe et al., 2006; Shannon-Lowe and Rowe, 2011; Nanbo et al., 2016), suggesting that cell-to-cell contact-mediated viral transmission is a dominant mode for infection of EBV in epithelial cells.

Previously we established an assay to assess the efficiency of EBV transmission mediated by cell-to-cell contact by coculturing EBV-infected BL cells and EBV-negative epithelial cells including human GC and NPC cell lines. By use of this assay, we demonstrated that direct cell contact induces bi-directional cell signaling pathways in cocultured cells, which leads to induction of the viral lytic cycle in BL cells and the subsequent enhancement of viral transmission into epithelial cells (Nanbo et al., 2012). We also observed that cocultivation enhanced the trafficking of adhesion molecules to the cell surface in BL cells in a vesicle recycling-dependent manner, and clathrin-dependent endocytosis in recipient cells for establishment of efficient viral transmission (Nanbo et al., 2016). Moreover, the mechanism by which EBV is transmitted to epithelial cells is distinct from that of the virological synapse (VS) employed by retroviruses. These studies indicate that EBV-infected B cells that migrate in the epithelial stroma or intraepithelial space contribute to efficient viral transmission into epithelial cells *via* cell contact.

It is well established that cell contact-mediated intracellular communications are mediated by secreted factors, such as cytokines and chemokines, in addition to physical interactions in the process of the antigen presentation in the immune system (Griffiths et al., 2010; Martín-Cófreces et al., 2014). Immune responses are typically initiated by the formation of an immunological synapse (IS), which is a highly organized, tight cellular contact interface formed between antigen-presenting cells (APCs), and responder cells, such as T cells. IS provides a platform for the presentation of antigens in major histocompatibility complexes (MHC) on the surface of the APC to the responder cells. Cell contact facilitates localized and directional membrane trafficking in both APCs and the responder cells. In APC, cell contact upregulates localized membrane trafficking, which leads to distributions of antigens that are loaded on MHC molecules and cytokine secretions at the IS. In responder cells, upregulation of membrane trafficking results in local release of lytic granules and recycling of T cell receptors at the interface of the cell contact (Griffiths et al., 2010; Martín-Cófreces et al., 2014). Although we have previously proposed that EBV exploits host membrane trafficking machinery for its establishment of successful viral transmission (Nanbo et al., 2016), the roles of secreted factors in this process are poorly understood.

Previous studies demonstrated an effect of TGF-β1, which is the prototypic member of the TGF-β superfamily, on inducing the EBV lytic cycle in some BL cell lines and epithelial cell lines (di Renzo et al., 1994; Fahmi et al., 2000; Fukuda et al., 2001; Liang et al., 2002; Iempridee et al., 2011). This induction is mediated by up-regulation of EBV's latent-lytic switch BamHI Z fragment leftward open reading frame 1 (*BZLF1)* gene.

The TGF-β superfamily consists of pleotropic cytokines secreted from a diverse range of cell types that regulate various cellular processes such as proliferation, differentiation, apoptosis, cell migration, cell adhesion, and immune responses (Massagué, 2012). The TGF-β signaling pathway is initiated by the binding of the TGF-β family ligands to TGF-β type II receptor (TβRII), which originally forms homo-dimers at the cell surface (Gilboa et al., 1998). The ligand-receptor complex facilitates the recruitment of homo-dimeric TGF-β type I receptor (TβRI) to form a hetero-tetrameric receptor complex (Feng and Derynck, 1996). The complex is then stabilized, which facilitates phosphorylation of TβRI (Gilboa et al., 1998). Phosphorylated TβRI mediates the activation of the transcription factors, the Smad proteins (Zhang et al., 1996). Upon activation of TGF-β signaling, Smad2 and Smad3 transiently associate with the TβR complex. These receptor-activated Smads then interact with Smad4 and translocate into the nucleus where target genes are transcriptionally activated (Massagué, 2012).

In the present study, we have found that cocultivation increased the lytic cycle in B cells and subsequent viral transmission into epithelial cell lines derived from GC in a time-dependent manner. The blockade of TGF-β signaling by use of a blocking antibody against TGF-β suppressed EBV transmission. Moreover, cell-contact facilitates the expression of TGF-β receptors on the surface of B cells and increases their susceptibility to TGF-β. Finally, we confirmed the secretion of TGF-β from the epithelial cells. The possible role of secretion factors in cell-to-cell contact-mediated EBV transmission is discussed.

## Materials and methods

### Cell culture and transfection

EBV-positive African BL-derived Akata^+^ cells (Takada et al., 1991) and Mutu I cells (Gregory et al., 1990), and EBV-negative Akata^−^ cells (Shimizu et al., 1994) were maintained in RPMI-1640 medium containing 10% fetal bovine serum (FBS) (Sigma-Aldrich, St. Louis, USA) and antibiotics. Akata^−^ EBV-eGFP cells are latently infected with a recombinant Akata strain EBV encoding eGFP gene inserted into viral BXLF1 ORF (EBV-eGFP) (Maruo et al., 2001a; Nanbo et al., 2012, 2016). Akata lymphoblastoid cell line (LCL) was generated by transformation of the B lymphocytes within the peripheral blood lymphocyte population by Akata EBV strain (Yajima et al., 2005). Akata^−^ EBV-eGFP cells and Akata LCLs were maintained in RPMI-1640 medium containing 10% FBS, antibiotics and 800 μg/ml G418 (Wako pure chemical industries Ltd., Osaka, Japan). The EBV-negative human GC epithelial AGS (Barranco et al., 1983; Yoshiyama et al., 1997), NU-GC-3 cells (Akiyama et al., 1988; Yoshiyama et al., 1997; Nishikawa et al., 1999; Oda et al., 2000; Maruo et al., 2001b; Iwakiri et al., 2003), and human embryonic kidney HEK293T cells were grown in high-glucose Dulbecco's modified Eagle's medium (DMEM) containing 10% FBS and antibiotics. Cells were maintained at 37°C in 5% CO_2_. Transfection of expression plasmids and siRNAs were carried out with TransIT-LT1 (Mirus, Madison, WI) and TransIT-TKO (Mirus), respectively.

### EBV-transmission assay

An EBV-transmission assay was conducted as described previously (Nanbo et al., 2012, 2016). Briefly, AGS cells or NU-GC-3 cells (5 × 10^4^) were cocultured with Akata^−^ EBV-eGFP cells (5 × 10^5^) for various times in 24-well plates. To remove the cocultured Akata^−^ EBV-eGFP cells, individual epithelial cells were washed with the medium three times, trypsinized, and cultured in 6-well plates for 6 h. Epithelial cells were harvested and fixed in 4% paraformaldehyde (PFA) in PBS for 10 min at room temperature. The percentages of eGFP-positive epithelial cells were analyzed by means of flow cytometry (FACSCalibur, Becton, Dickinson and company, San Diego, USA). In parallel with flow cytometric analysis, the same sample was analyzed by a confocal laser scanning microscope to confirm that the sample did not contain Akata^−^ EBV-eGFP cells. To examine the effect of exosomes secreted from B cells, epithelial cells were pretreated with 0.25 μg/ml purified exosomes for 3 days and subsequently cocultured with Akata^−^ EBV-eGFP cells for 2 days. To investigate the effect of downregulation of Rab27a in B cells on viral transmission, Akata^−^ EBV-eGFP cells in which Rab27a expression was downregulated were cocultured with epithelial cells for 5 days. To assess the effect of down-regulation of Rab27a in epithelial cells on viral transmission, Akata^−^ EBV-eGFP cells were cocultured for 5 days with epithelial cells in which Rab27a expression was downregulated. To examine the effect of the blocking antibody for TGF-β in EBV transmission, Akata^−^ EBV-eGFP cells and epithelial cells were cocultured for 5 days in the presence of the mouse monoclonal antibody for TGF-β1, β2, and β3 (clone 1D11) (R&D SYSTEMS, Minneapolis, USA) (Akool El et al., 2008). EBV transmission was analyzed as described above. The flow cytometric analysis was performed with three biological replicates independently and the results were statistically analyzed by means of the Student's *t*-test.

### Immunofluorescence staining

For analysis of induction of the lytic cycle, Akata^−^ EBV-eGFP cells (1 × 10^6^) were cocultured with AGS cells or NU-GC-3 cells (1 × 10^6^) in 10 cm plates with or without trans-well inserts (Corning, Toledo, USA) for various times. The cells were fixed with 4% PFA in PBS for 10 min at room room temperature, permeabilized in PBS containing 0.05% Triton X-100 for 10 min at room temperature and blocked in PBS containing 1% bovine serum albumin (BSA) for 20 min at room temperature. The cells were incubated with a mouse anti-gp350 monoclonal antibody (clone C-1) (Thorley-Lawson and Geilinger, 1980) for 1 h at room temperature. After being washed twice in PBS, the cells were incubated with Alexa Fluor 488-labeled secondary antibody (Thermo Fisher Scientific, Waltham, USA) for 1 h at room temperature. After washing, the nuclei were counterstained with Hoechst 33342 (Cell Signaling Technology, Trask Lane, USA). Images were collected with a 60 × water-immersion objective (NA = 1.3) of a confocal laser scanning microscope (Fluoview FV10i, Olympus, Tokyo, Japan) and acquired by using FV10-ASW software (Olympus). Four fields containing approximately 100 cells were randomly collected, and the fractions of gp350-positive B cells were measured.

### Analysis of expression of TGF-β receptors in akata cells

To determine the expression of TGF-β receptors in BL cells, Mutu I, Akata^−^ EBV-eGFP cells, Akata^−^, or Akata^+^ (1 × 10^6^ cells) were incubated with the rabbit anti-TβRI or -TβRII polyclonal antibody (1:100 dilution, Abcam, San Francisco, USA) for 1 h at 4°C in PBS containing 2% BSA. After being washed twice, the cells were incubated with Alexa Fluor 488-conjugated secondary antibody (1:1,000-dilution) for 1 h at 4°C in the same buffer. After being washed, the expression of the receptors was analyzed by means of flow cytometry. The transcript-level of expression of TGF-β receptors in BL cells was analyzed by RT-PCR with sense (CGTGCTGACATCTATGCAAT) and antisense (AGCTGCTCCATTGGCATAC) for TβRI, and with sense (CAGTTTGCCATGACCCCAAG) and antisense (CATTGCACTCATCAGAGCTACAGG) for TβRII.

### Analysis of expression of ephrin receptor A2 in epithelial cells

To determine the expression of ephrin receptorA2 (EphA2) in epithelial cells, AGS, NU-GC-3 cells (1 × 10^6^ cells) were incubated with a rabbit anti-EphA2 monoclonal antibody (1:200 dilution, Cell Signaling Technology) for 1 h at 4°C in PBS containing 2% BSA. After being washed twice, the cells were incubated with Alexa Fluor 488-conjugated secondary antibody (1:1,000-dilution) for 1 h at 4°C in the same buffer. After being washed, the expression of the receptors was analyzed by means of flow cytometry.

### Enzyme-linked immunosorbent assay (ELISA)

AGS cells were grown with or without Akata^−^ EBV-eGFP cells for 5 days and the supernatants of cultured cells were harvested. The amount of TGF-β in the supernatants was analyzed by a human TGF-β ELISA Kit (Abcam) in accordance with the manufacturer's instruction.

### Purification of exosomes

For the purification of exosomes, Akata^−^, Akata^+^, Akata-LCLs (1 × 10^7^ cells, each) were grown in RPMI 1640 medium containing 10% exosome-depleted FBS, which was prepared by centrifugation at 112,400 × g for 4 h at 4°C with an SW28 rotor (Beckman Fullerton, USA). Culture medium containing exosomes was harvested and centrifuged at 1,500 × g for 10 min and at 6,000 × g for 20 min to remove cells and cell debris, respectively. The exosomes were pelleted by centrifugation at 112,400 × g for 1 h at 4°C with an SW28 rotor (Beckman). The pelleted exosomes were resuspended in TNE buffer [10 mM Tris-HCl (pH 7.6), 100 mM NaCl, 1 mM EDTA] overnight. The fractions containing exosomes were confirmed by western blot analysis with anti-CD63 monoclonal antibody (clone MEM-250, Abnova, Taipei, Taiwan).

### Knockdown of Rab27a by shRNA

To knock down Rab27a gene expression in Akata^−^ EBV-eGFP, AGS, and NU-GC-3 cells, the cells were transduced with an HIV-based lentiviral pLKO.1 vector of a shRNA encoding the corresponding target sequence 5′-TATACAATTCAAATGCACAGG-3′ (GE healthcare Dharmacon Inc., Lafayette, USA). As a control, a pLKO.1 plasmid encoding a sequence that does not target any known genes (Sigma-Aldrich) was used. Recombinant lentiviruses were generated by co-transfection of pLKO.1 vector, pCAGGS-HIV gag/pol, pCAGGS-Rev, and pCMV-VSV-G in HEK293T cells. The culture medium was collected at 48 and 72 h.p.t., and concentrated 100-fold by ultracentrifugation at 135,500 × g for 2 h at 4°C with an 80Ti rotor (Beckman). For lentiviral infections, Akata^−^ EBV-eGFP, AGS, and NU-GC-3 cells (1 × 10^5^) were grown in 24-well plates, the culture medium was replaced with ice-cold DMEM supplemented with 10% FBS and 20 mM HEPES (pH 7.4), and the cells were incubated with viral stocks for 1 h at 4°C at a multiplicity of infection (m.o.i.) of 5. After being washed twice with complete medium, the cells were cultured in complete medium for 48 h and used for further analyses. Rab27a expression in the harvested cells was confirmed by real time RT-PCR as described before (Nawandar et al., 2017). The total RNA extracted with a RNeasy Kit (Qiagen, Hilden, Germany) was used for reverse transcription using oligo(dT) primer and AMV reverse transcriptase (RT) (Roche, Basel, Switzerland). Real-time PCR was performed on the reverse-transcribed cDNA by using iTaq Universal SYBR green mix (Bio-Rad, Hercules, USA) with sense (GAAGCCATAGCACTCGCAGAG) and antisense (ATGACCATTTGATCGCACC) oligonucleotides. As an internal control, RNA encoding the TATA-BOX protein (TBP) was amplified by use of sense (AAATATTGTATCCACAGTGAATCTTGGTTG) and antisense (GAACTGAAAATCAGTGCCGTGGTTCGTGGC) oligonucleotides. The reactions were incubated at 96°C for 2 min followed by 40 cycles of 96°C for 30 s and 60°C for 30 s. Data were collected on a Bio-Rad CFX96 instrument (Bio-Rad). Transcripts were quantified using the ΔΔCq method.

### Knockdown of Eph2A and TGF-β by siRNA

To knock down Eph2A or TGF-β gene expression in epithelial cells, AGS cells (1 × 10^5^) were transfected with siRNA encoding the corresponding target sequences as follows: 5′-GCAGCAAGGUGCACGAAUU-3′ (siEph2A#1); 5′-GCAGCAAGGUGCACGAAUU-3′ (siEph2A#2) (Zhang et al., 2018); 5′-GGCUCAAGUUAAAAGUGGvAUU-3′ (siTGF-β) (Thermo Fisher Scientific). As a control, a siRNA encoding a sequence that does not target any known genes (Thermo Fisher Scientific) was used. The cells were harvested at 72 h.p.t. Eph2A expression in the harvested cells was confirmed with flow cytometry with a rabbit anti-Eph2A monoclonal antibody (1:1,000-dilution, Cell Signaling Technology). The amounts of TGF-β in the cell culture media was analyzed by ELISA.

### Quantification of EBV titer by real-time PCR

To determine the effect of downregulation of Rab27a in Akata^−^ EBV-eGFP cells on release of progeny EBV, the cells in which Rab27a was downregulated were treated with 1% goat anti-human IgG (αhIgG) (DAKO, Glostrup, Denmark) for 48 h. The supernatant was treated with 5 U DNase at 37°C for 15 min to obtain only encapsidated EBV DNA. DNase was inactivated by incubation at 70°C for 5 min in the presence of 2.5 mM EDTA. EBV DNA in the supernatant was isolated using DNeasy Blood and Tissue Kit (Qiagen). For detection of EBV DNA, real-time PCR was carried out as previously described (Vereide and Sugden, 2011) with sense (CGGAAGCCCTCTGGACTTC) and antisense (CCCTGTTTATCCGATGGAATG) oligonucleotides and probe (TGTACACGCACGAGAAATGCGCC) specific for the EBV-encoded *BARF5* sequence. As an internal control, cellular Rhodopsin was analyzed using sense (ATCAGGAACCATTGCCACGTCCTA) and antisense (AGGCCAAAGATGGACACACAGAGT) oligonucleotides and probe (AGCCTCTAGTTTCCAGAAGCTGCACA).

## Results

### Time-dependent cell-to-cell contact-mediated EBV transmission

We have previously established an assay to assess the efficiency of cell-to-cell transmission of EBV by coculturing latently EBV-infected BL cells with EBV-negative human epithelial cells (Nanbo et al., 2012, 2016). We used the BL-derived Akata^−^ EBV-eGFP cells as virus donor cells, which are latently infected with a recombinant Akata-derived strain of EBV encoding eGFP (Maruo et al., 2001b). After coculturing with the EBV-negative human GC AGS and NU-GC-3 cells for various times, the transmission of EBV-eGFP into the epithelial cells was analyzed by quantifying the percentage of eGFP-positive cells with a confocal laser scanning microscope and flow cytometry.

**Figure 1:**
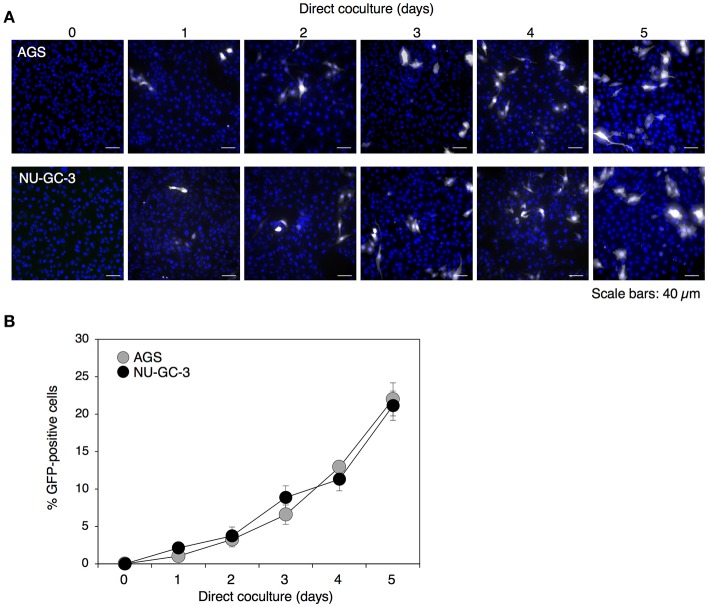
Time-dependent enhancement of cell-to-cell contact-mediated EBV transmission into epithelial cells. **(A)** Analysis of cell-to-cell contact-mediated EBV transmission into epithelial cells with a confocal laser scanning microscope. Akata^−^ EBV-eGFP cells were cocultured with AGS (top) or NU-GC-3 cells (bottom) for various times. eGFP-positive, infected epithelial cells (white) were visualized by a confocal laser scanning microscope. The nuclei were counterstained with Hoechst 33342 (blue). Representative images are shown. Scale bars: 40 μm. **(B)** Summary of cell-to-cell contact-mediated EBV transmission. Akata^−^ EBV-eGFP cells were cocultured with AGS (gray) or NU-GC-3 cells (black) for various times. The percentages of eGFP-positive, infected epithelial cells were analyzed by means of flow cytometry. The experiment was performed three times independently and the average values and their SD are shown for each condition.

Previously we have observed that EBV-eGFP infected approximately 2 or 5% of populations of AGS or NU-GC-3 cells, respectively, by the coculturing directly with Akata^−^EBV-eGFP for 48 h (Nanbo et al., 2012, 2016). In this study, we analyzed viral transmission after 5 days of direct cocultivation. We observed that the transmission of eGFP-positive AGS and NU-GC-3 cells increased in a time-dependent manner and approximately 20% of epithelial cells were infected with EBV-eGFP after 5 days of cocultivation (Figures 1A,B).

### Cocultivation with epithelial cells induces lytic cycle in B cells

**Figure 2:**
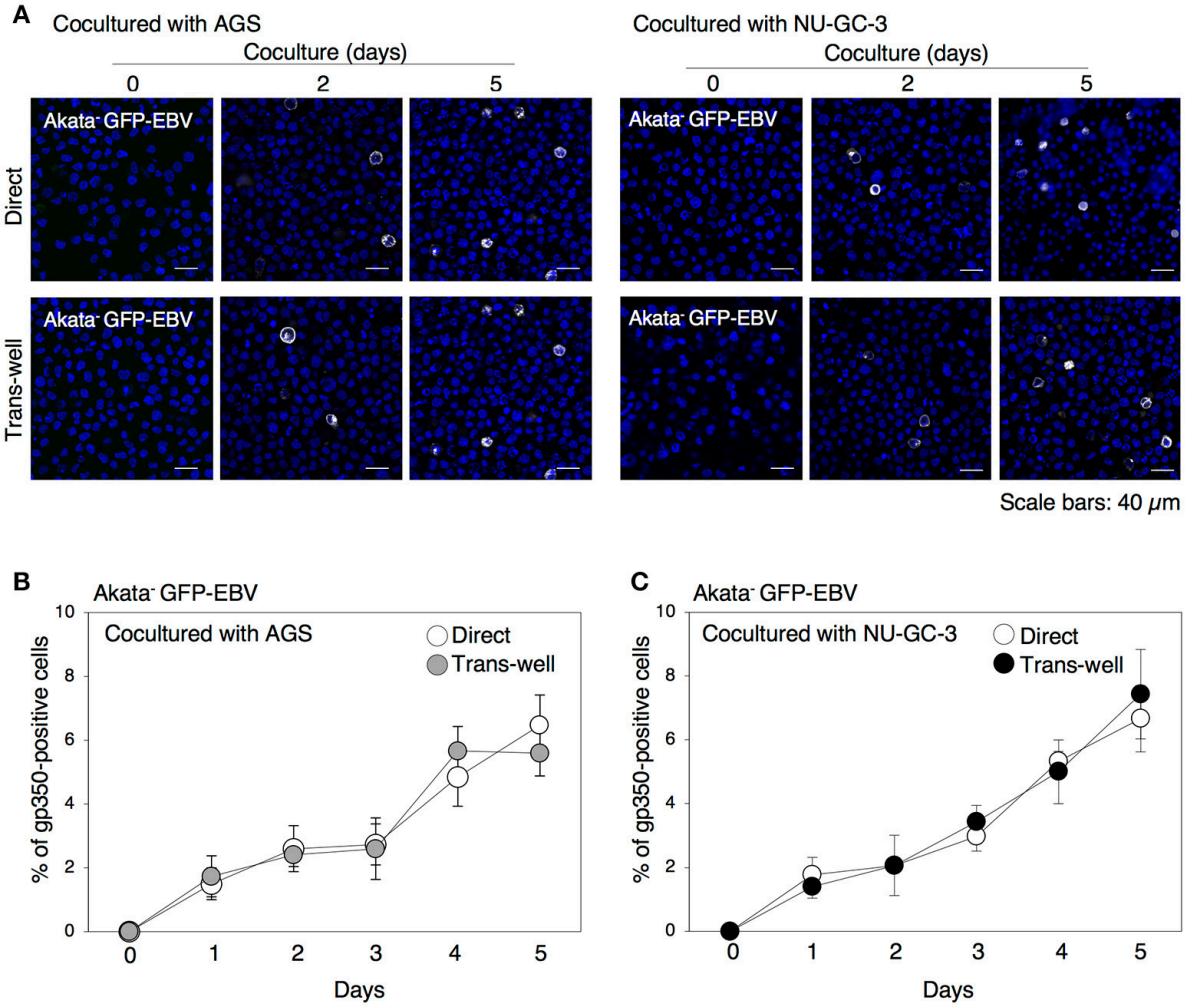
Time-dependent induction of EBV replication in B cells cocultured with epithelial cells. **(A)** gp350 expression in B cells cocultured with epithelial cells. Akata^−^ EBV-eGFP cells were cocultured with AGS (left) or NU-GC-3 cells (right) with (bottom) or without (top) trans-well inserts for various times. B cells were harvested and expression of gp350 (white) was analyzed by immunofluorescence staining. The nuclei were counterstained with Hoechst 33342 (blue). Representative images are shown. Scale bars: 40 μm. **(B,C)** Summary of expression of gp350 in B cells cocultured with epithelial cells. Akata^−^ EBV-eGFP cells were cocultured with AGS **(B)** and NU-GC-3 cells **(C)** with (gray and black, respectively) or without (white) trans-well inserts for various times. Percentage of gp350-positive cells was analyzed by immunofluorescence staining. The experiment was performed three times independently and the average values and their SD are shown for each condition.

We have previously demonstrated that 24 h-long cell contact with epithelial cells initiates the lytic cycle in approximately 2% of Akata^−^ EBV-eGFP (Nanbo et al., 2012). In this study, we further examined the effect of long-term cocultivation on induction of lytic cycle in BL cells by immunofluorescence staining for gp350, which is a major viral glycoprotein expressed in the later stage of the lytic cycle (Longnecker and Cohen, 2013). Consistent with previous studies showing that viral replication is spontaneously initiated in only a limited fraction of EBV-positive Akata cells (Takada and Ono, 1989), no or limited gp350-positive cells were detected without cell contact (Figures 2A–C). We observed that direct cocultivation with epithelial cells increased the ratio of gp350-positive B cells in a time-dependent manner. Approximately 5% of cell population underwent the lytic cycle by 5 days of cocultivation (Figures 2A–C). We also observed that cocultivation through a trans-well similarly induced viral replication in B cells, indicating that physical cell contact is not required for enhancement of viral replication in B cells.

### Blockage of TGF-β signaling suppressed cell-to-cell contact-mediated EBV transmission

**Figure 3:**
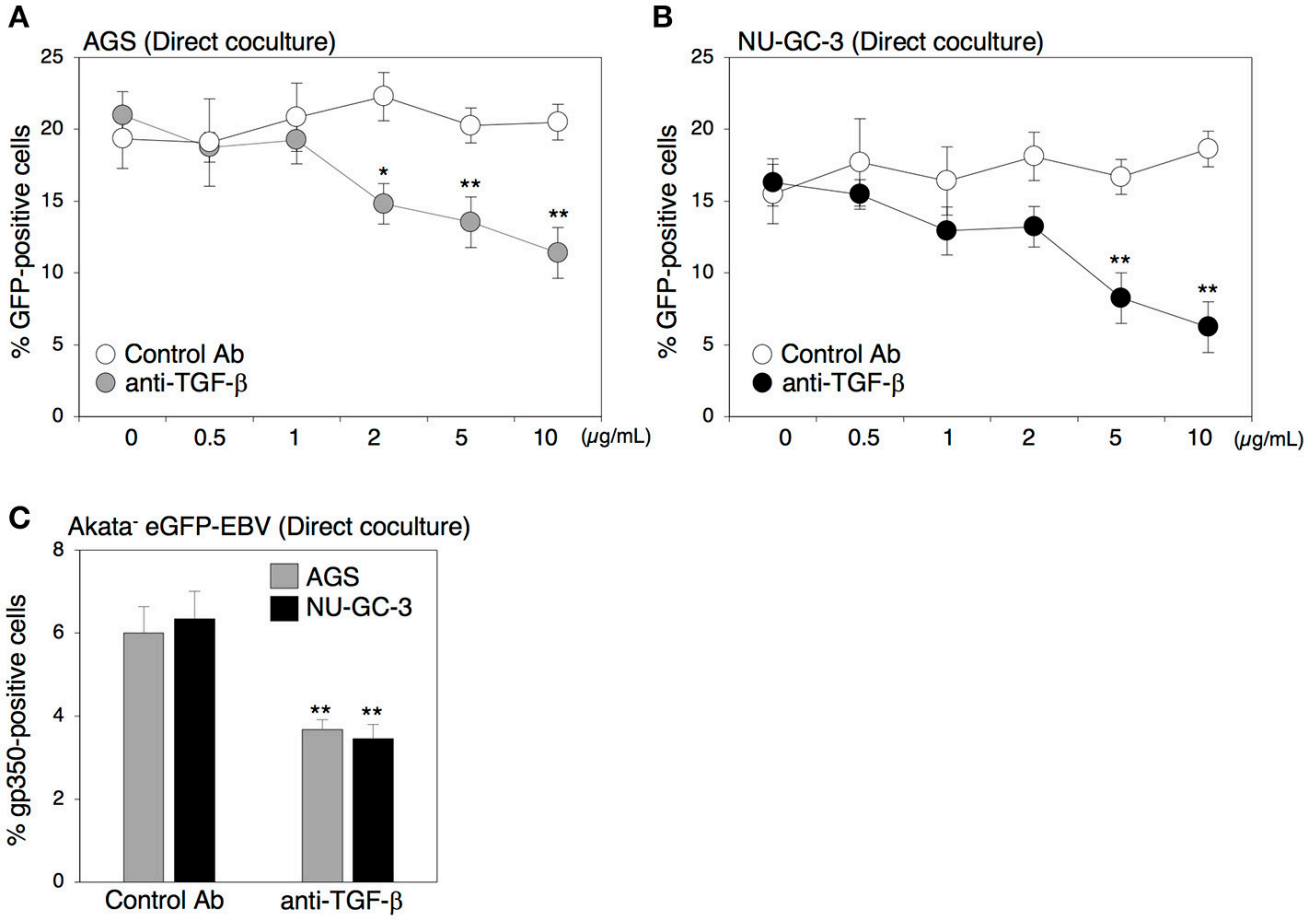
Effect of the blocking antibody for TGF-β on cell-to-cell contact-mediated EBV transmission. **(A,B)** Effect of the blocking antibody for TGF-β on cell-to-cell contact-mediated EBV transmission. Akata^−^ EBV-eGFP cells were cocultured with AGS cells **(A)** or NU-GC-3 cells **(B)** for 5 days in the presence of the blocking antibody for human TGF-β (0.5, 1.0, 2.0, 5.0, 10.0 μg/ml). As a control, an antibody for ER was used. The percentages of eGFP-positive, infected epithelial cells were analyzed by means of flow cytometry. The experiment was performed three times independently and the average and its SD are shown in each condition. ^*^P < 0.05; ^**^P < 0.01 vs. respective control (Student's *t*-test). **(C)** Effect of the blocking antibody for TGF-β on induction of lytic cycle in cocultured B cells. Akata^−^ EBV-eGFP cells were cocultured with AGS (gray) or NU-GC-3 cells (black) for 5 days in the presence of 10.0 μg/ml anti-human TGF-β. As a control, an anti-estrogen receptor (ER) antibody was used. B cells were harvested, and percentage of gp350-positive cells was analyzed by immunofluorescence staining. The experiment was performed three times independently and the average values and their SD are shown for each condition. ^**^P < 0.01 vs. respective control (Student's *t*-test).

Both direct and indirect cell contact induced viral replication in B cells (Figure 2), suggesting that secreted factors derived from cocultured cells contribute to induction of the lytic cycle in B cells. Because TGF-β1 induces the lytic cycle in some BL cells (di Renzo et al., 1994; Fahmi et al., 2000; Fukuda et al., 2001; Liang et al., 2002; Iempridee et al., 2011), we further assessed the role of TGF-β1 in EBV transmission by means of a blocking antibody against TGF-β family. The blocking antibody strikingly suppressed EBV transmission in a dose-dependent manner (Figures 3A,B). Moreover, direct coculture-mediated lytic cycle induction in B cells was also suppressed in the presence of the same blocking antibody (Figure 3C), suggesting that TGF-β secreted from epithelial cells promotes viral replication.

### Direct cell-contact facilitates the expression of TGF-β receptors on the surface of B cells and increases their susceptibility to TGF-β secreted from epithelial cells

**Figure 4:**
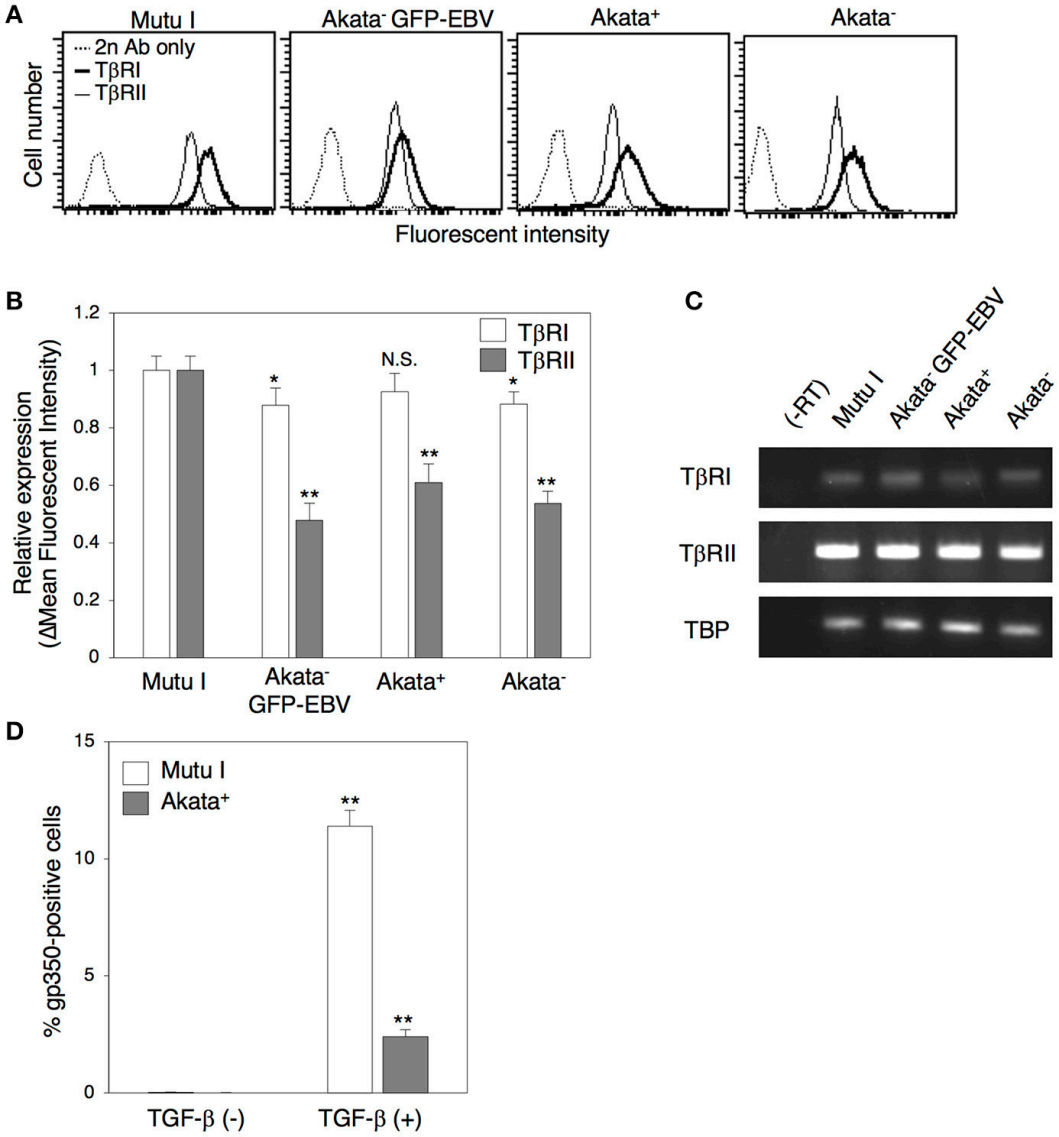
Expression of TGF-β receptors in Akata cells. **(A)** Flow cytometric analysis for expression of TGF-β receptors in BL cells. Expression of TβRI (boldface lines) or TβRII (thin lines) in Mutu I, Akata^−^ GFP-EBV, Akata^+^, or Akata^−^ cells was analyzed by means of flow cytometry. Representative histograms are shown. As a control, cells were incubated with the secondary antibody alone (dotted lines). **(B)** Summary of expression of TGF-β receptors in BL cells. The relative expression of TβRI (white) or TβRII (gray) in Mutu I, Akata^−^ GFP-EBV, Akata^+^, or Akata^−^ cells was analyzed by flow cytometry. The data were normalized to the expression of TGF-β receptors in Mutu I cells. The experiment was performed three times independently, and the averages and SD are shown for each condition. ^*^P < 0.05; ^**^P < 0.01 vs. respective control; N.S., not significant (Student's *t*-test). **(C)** mRNA expression of TGF-β receptors in BL cells. Total RNA was isolated from Mutu I, Akata^−^ GFP-EBV, Akata^+^, or Akata^−^ cells and the expression of mRNA of TβRI (white) and TβRII (gray) was analyzed by RT-PCR. As an internal control, the expression of the TATA-binding protein (TBP) mRNA was analyzed. **(D)** Induction of lytic cycle in Akata cells by treatment of TGF-β. Mutu I (white) or Akata^−^ GFP-EBV (gray) cells were treated with a recombinant TGF-β 100 pg/ml) for 48 h. B cells were harvested and the percentage of gp350-positive cells was analyzed by immunofluorescence staining. The experiment was performed three times independently and the average values and their SD are shown for each condition. ^**^P < 0.01 vs. respective control (Student's *t*-test).

Previous studies demonstrated that some BL cells including Akata cells exhibit a defect in TGF-β-mediated induction of lytic cycle, because of a lack of expression of TβRII (Inman and Allday, 2000; Fukuda et al., 2006; Iempridee et al., 2011), which appears to be mediated by epigenetic silencing (Di Bartolo et al., 2008). Thus, we analyzed the expression of TGF-β receptors in Akata cells by means of flow cytometry. Consistent with previous reports (Inman and Allday, 2000; Fukuda et al., 2006; Iempridee et al., 2011), the type I latently EBV-infected BL cell line, Mutu I (Gregory et al., 1990), expressed both TβRI and TβRII (Figures 4A,B). We observed that Akata^−^ eGFP-EBV, Akata^+^ and Akata^−^ clones were also TβRI and TβRII-positive, although their expression levels were lower than those in Mutu I cells (Figures 4A,B). We confirmed the expression of mRNA for both receptors in Akata clones by RT-PCR (Figure 4C). Treatment of Akata^−^ eGFP-EBV with a recombinant TGF-β induced the lytic cycle, indicating that TβRI and TβRII expressed in Akata cells are functional (Figure 4D).

**Figure 5:**
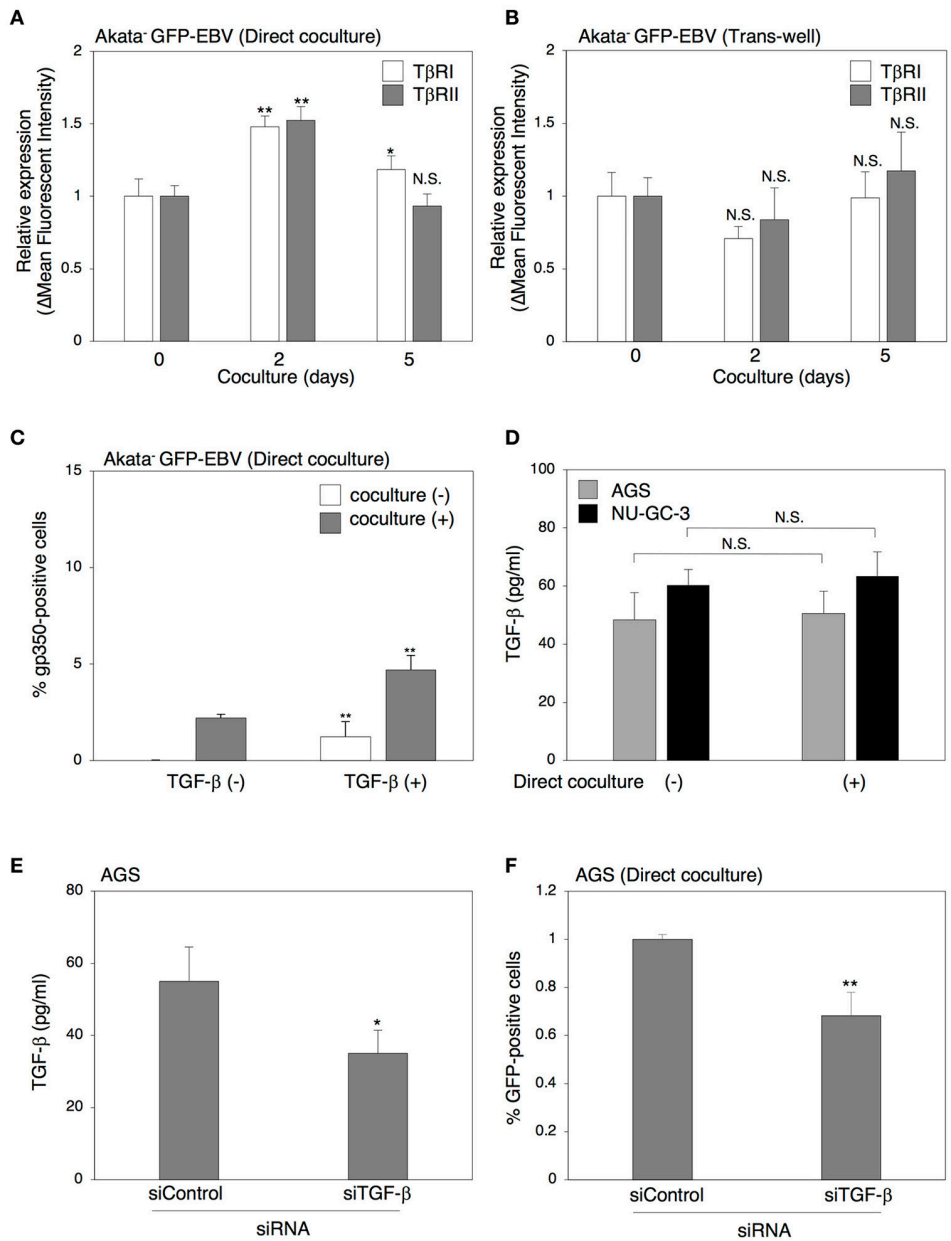
Direct cell-contact facilitates the expression of TGF-β receptors on the surface of B cells and their susceptibility to TGF-β. **(A,B)** Effect of cell contact on expression of TGF-β receptors in B cells. Akata^−^ EBV-eGFP cells were cocultured with AGS cells directly **(A)** or through a trans-well **(B)** for 2 or 5 days. B cells were harvested and the expression of TβRI (white) and TβRII(gray) was analyzed by means of flow cytometry. The data were normalized to their expression in Akata^−^ EBV-eGFP without cocultivation. The experiment was performed three times independently and the average values and their SD are shown for each condition. ^*^P < 0.05; ^**^P < 0.01 vs. respective control; N.S., not significant (Student's *t*-test). **(C)** Effect of cell contact on TGF-β-mediated induction of lytic cycle in Akata cells. Akata^−^ GFP-EBV cells were cocultured with (gray) or without (white) AGS cells for 2 days. B cells were harvested and treated with a recombinant TGF-β (100 pg/ml) for 48 h. The percentage of gp350-positive cells was analyzed by immunofluorescence staining. The experiment was performed three times independently and the average values and their SD are shown for each condition. ^**^P < 0.01 vs. respective control (Student's *t*-test). **(D)** The amount of TGF-β released from cocultured epithelial cells. AGS (gray) or NU-GC-3 (black) cells were cultured with or without Akata^−^ EBV-eGFP cells for 5 days and the supernatants of cultured cells were harvested. The amount of TGF-β in the supernatants was analyzed by ELISA. The experiment was performed three times independently and the average values and their SD are shown for each condition. N.S.; not significant (Student's *t*-test). **(E)** Downregulation of TGF-β by siRNA. Total RNA was isolated from AGS cells at 72 h-post transfection of control siRNA or siRNA against TGF-β. Downregulation of expression for TGF-β was analyzed by ELISA. The experiment was performed three times independently and the average and its SD are shown in each condition. ^*^P < 0.05 vs. respective control (Student's *t*-test). **(F)** Effect of downregulation of TGF-β in AGS cells on cell-to-cell contact-mediated EBV transmission. Akata^−^ EBV-eGFP cells were cocultured with AGS cells, in which TGF-β was downregulated for 5 days. The percentages of eGFP-positive, infected epithelial cells were analyzed by means of flow cytometry. The experiment was performed three times independently and the average and its SD are shown in each condition. ^**^P < 0.01 vs. respective control (Student's *t*-test).

We next investigated the effect of cell contact on the TGF-β signaling pathway. Two days of direct cocultivation upregulated the expression of both TβRI and TβRII on the surface of Akata^−^ eGFP-EBV (Figure 5A). In contrast, cocultivation through a trans-well did not upregulate the expression of TGF-β receptors (Figure 5B). We also observed that direct cocultivation with epithelial cells for 2 days increased the TGF-β-induced lytic cycle in Akata^−^ eGFP-EBV (Figure 5C). All these results indicate that functional TGF-β receptors were expressed in Akata cells and their expression was enhanced by direct cocultivation with epithelial cells, which resulted in the increase of their susceptibility to TGF-β. We then tested whether TGF-β is produced from epithelial cells directly cocultured with B cells. An ELISA assay revealed that cell culture supernatants of AGS cells cultured for 5 days contained TGF-β1 and its secretion was not affected by direct cocultivation with B cells (Figure 5D), indicating that TGF-β can be spontaneously secreted from epithelial cells. In contrast, the TGF-β in cell culture supernatants of B cells was undetectable (data not shown). We further confirmed the role of TGF-β in cell-to-cell contact-mediated viral transmission by cocultivation of Akata^−^ EBV-eGFP with AGS cells in which TGF-β expression was downregulated (Figure 5E). Downregulation of TGF-β in AGS cells decreased viral transmission (Figure 5F). These data indicate that direct cell-contact facilitates the expression of TGF-β receptors on the surface of B cells and increases their susceptibility to TGF-β secreted from epithelial cells.

### Exosomes released from cocultured cells partly contribute to viral transmission

**Figure 6:**
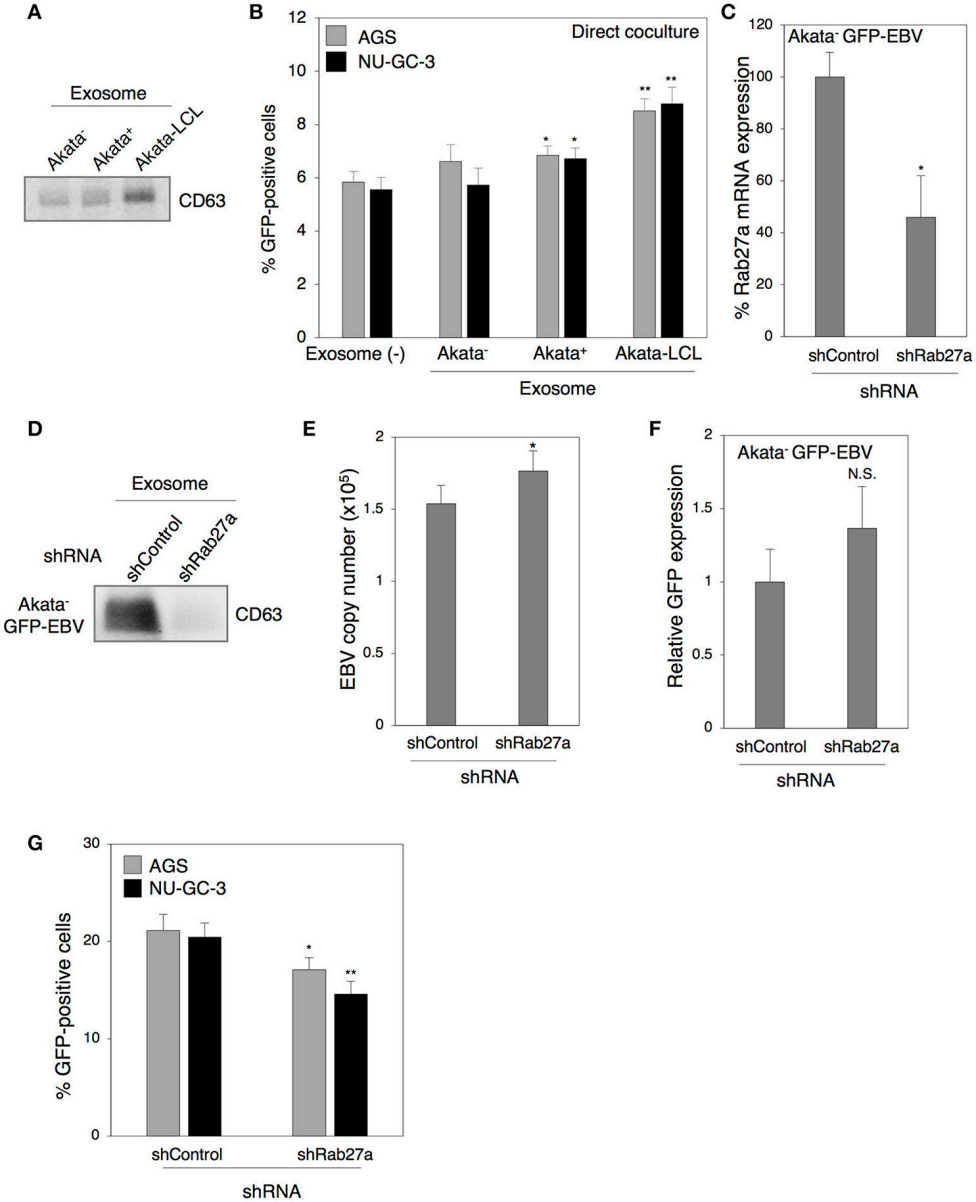
Role of exosomes released from B cells in cell-to-cell contact-mediated EBV transmission. **(A)** Purified exosomes derived from B cells. Exosomes were purified from culture medium of Akata^−^ (1st lane), Akata^+^ (2nd lane), and Akata-LCL (3rd lane) cells. Four micrograms of exosomes were analyzed by Western blotting with anti-CD63 monoclonal antibody. **(B)** The effect of exosomes released from B cells on cell-to-cell contact-mediated EBV transmission. AGS (gray) or NU-GC-3 cells (black) were pre-treated with or without exosomes released from Akata^−^, Ataka^+^ or Akata-LCL for 3 days. Cells were washed and cocultured with Akata^−^ EBV-eGFP cells for 48 h. The percentages of eGFP-positive, infected epithelial cells were analyzed by means of flow cytometry. The experiment was performed three times independently and the average and its SD are shown in each condition. ^*^P < 0.05; ^**^P < 0.01 vs. respective control; N.S., no significant (Student's *t*-test). **(C)** Downregulation of Rab27a by shRNA. Total RNA was isolated from Akata^−^ EBV-eGFP cells transiently expressing shRNA against Rab27a. Downregulation of mRNA expression for Rab27a was analyzed by quantitative RT-PCR. The data were normalized to the cells expressing a control shRNA. The experiment was performed three times independently and the average and its SD are shown in each condition. ^*^P < 0.05 vs. respective control (Student's *t*-test). **(D)** Effect of downregulation of Rab27a on the release of exosomes. Exosomes released from Akata^−^ EBV-eGFP cells transiently expressing control shRNA (1st lane) or shRNA for Rab27a (2nd lane) was purified. Four micrograms of exosomes were analyzed by Western blotting with anti-CD63 monoclonal antibody. **(E)** Effect of downregulation of Rab27a on release of EBV. Akata^−^ EBV-eGFP cells transduced with the lentivirus encoding shRNA for Rab27a. Forty-eight hour post-infection, the cells were treated with 1% anti-human IgG for 48 h. EBV titers in the supernatants were analyzed by quantitative PCR. As an internal control, the human rhodopsin gene was used. The experiment was performed three times independently and the average and its SD are shown in each condition. ^*^P < 0.05 vs. respective control (Student's *t*-test). **(F)** The effect of downregulation of Rab27a on GFP expression in Akata^−^ EBV-eGFP cells. Akata^−^ EBV-eGFP cells transduced with the lentivirus encoding shRNA for Rab27a. 48 h post-infection, the expression of GFP was analyzed by means of flow cytometry. N.S., no significant (Student's *t*-test). **(G)** The effect of downregulation of Rab27a in B cells on cell-to-cell contact-mediated EBV transmission. Akata^−^ EBV-eGFP cells in which Rab27a was downregulated by shRNA were cocultured with AGS cells (gray) or NU-GC-3 cells (black) for 5 days. The percentages of eGFP-positive, infected epithelial cells were analyzed by means of flow cytometry. The experiment was performed three times independently and the average and its SD are shown in each condition. ^*^P < 0.05; ^**^P < 0.01 vs. respective control (Student's *t*-test).

In our previous study, we demonstrated that treatment with exosomes released from EBV-positive B cells upregulates ICAM-1 expression in EBV-negative epithelial cells (Nanbo et al., 2013). We have also shown that various adhesion molecules including ICAM-1 are involved in the establishment of cell-to-cell contact-mediated EBV transmission (Nanbo et al., 2016). We now examined the role of exosomes derived from B cells in this process. Exosomes were isolated from culture supernatants of EBV-negative and -positive Akata cells and purified by ultracentrifugation (Nanbo et al., 2013). Western blot revealed that purified exosome fractions contained CD63, a tetraspanin that is defined as a specific marker for exosomes (Figure 6A). Target cells were pretreated with individual exosomes for 3 days followed by 2 days of direct cocultivation with Akata^−^ EBV-eGFP cells. Viral transmission was moderately upregulated in the presence of exosomes released from Akata^+^ cells (Figure 6B). Since exosomes released from B cells in Type III latency induce ICAM-1 expression in the target cells more efficiently compared with that released from B cells in type I latency (Nanbo et al., 2013), the effect of exosomes released from Akata EBV-transformed LCL (Akata-LCL) in viral transmission was also examined. Exosomes released from Akata-LCLs promoted viral transmission more significantly compared with that derived from Akata^+^. To further examine the role of exosomes released from B cells in viral transmission, we knocked down the expression of Rab27a, a small GTPase, which contributes to exosome secretion *via* exocytosis of multi-vesicular bodies (MVB) (Ostrowski et al., 2010; Peinado et al., 2012), in Akata^−^ EBV-eGFP cells. We confirmed the downregulation of Rab27a mRNA expression in Akata^−^ EBV-eGFP cells by quantitative RT-PCR (Figure 6C). Western blot analysis revealed the suppression of exosome secretion from Akata^−^ EBV-eGFP in which Rab27a was downregulated (Figure 6D). To exclude the possibility that suppression of Rab27a-dependent exocytosis abrogates the release of progeny EBV, we measured the titer of EBV in the supernatant of Rab27a knockdown cells treated with anti-human IgG to induce the lytic cycle by cross-linking of surface IgG (Takada, 1984). The viral titers were slightly increased by downregulation of Rab27a expression (Figure 6E), indicating that blockage of exosome secretion pathway does not suppress EBV egress. We also confirmed the effect of downregulation of Rab27a on the expression of GFP reporter gene in transduced B cells. Knockdown of Rab27a exhibited little effect on GFP expression (Figure 6F). EBV transmission was moderately suppressed by direct cocultivation with Akata^−^ EBV-eGFP cells in which Rab27a expression was downregulated (Figure 6G).

**Figure 7:**
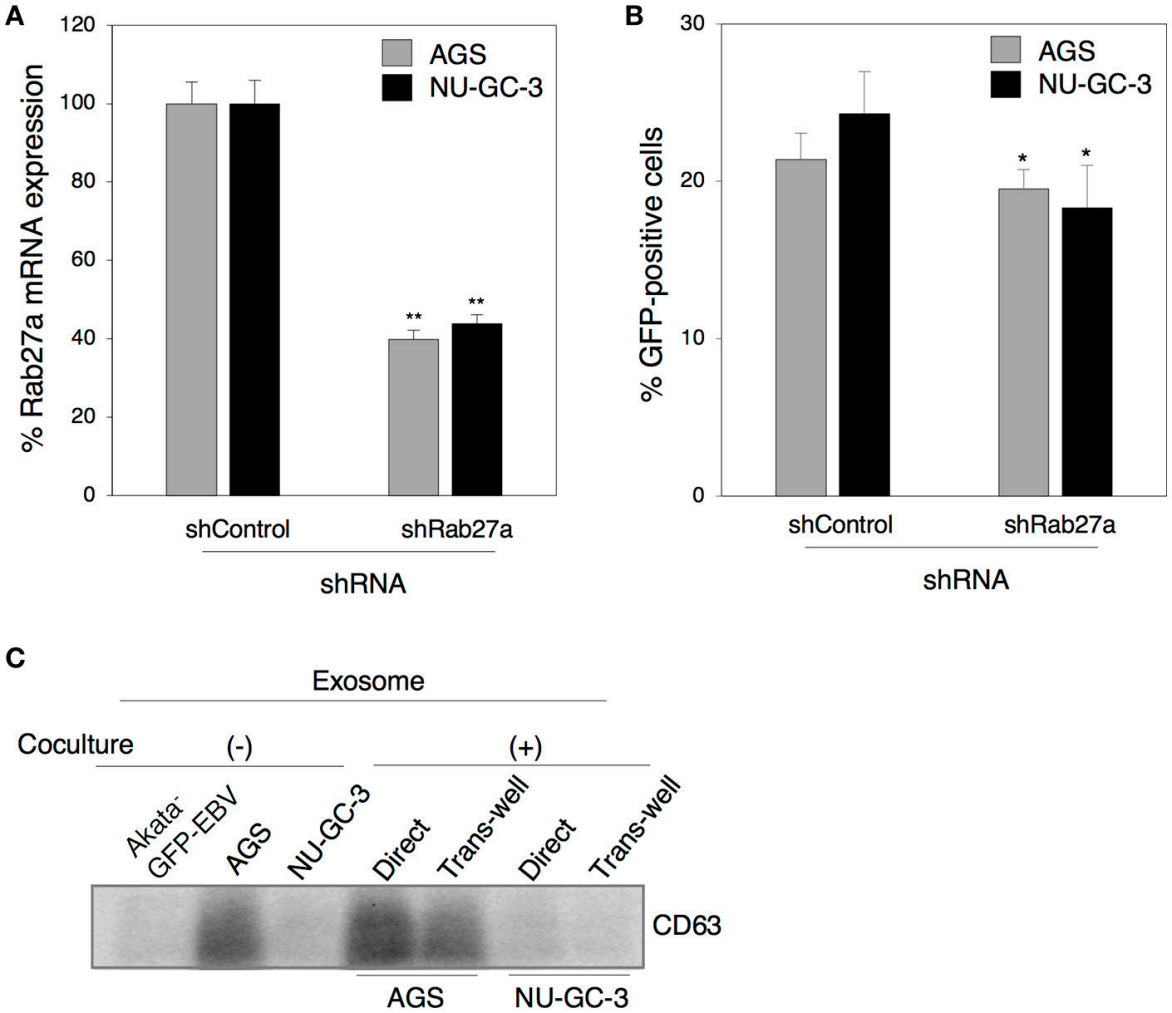
The role of exosomes released from epithelial cells in cell-to-cell contact-mediated EBV transmission. **(A)** Downregulation of Rab27a by shRNA. Total RNA was isolate from AGS cells (gray) or NU-GC-3 cells (black) transiently expressing shRNA against Rab27a. Downregulation of mRNA expression for Rab27a was analyzed by quantitative RT-PCR. The data were normalized to the cells expressing control shRNA. The experiment was performed three times independently and the average and its SD are shown in each condition. ^**^P < 0.01 vs. respective control (Student's *t*-test). **(B)** Effect of downregulation of Rab27a in epithelial cells on cell-to-cell contact-mediated EBV transmission. AGS cells (gray) or NU-GC-3 cells (black) in which Rab27a was downregulated by shRNA were cocultured with Akata^−^ EBV-eGFP for 5 days. The percentages of eGFP-positive, infected epithelial cells were analyzed by means of flow cytometry. The experiment was performed three times independently and the average and its SD are shown in each condition. ^*^P < 0.05 vs. respective control (Student's *t*-test). **(C)** The effect of cell contact on the release of exosomes. Exosomes released from Akata^−^ EBV-eGFP cells (1st lane), AGS cells (2nd lane), NU-GC-3 cells (3rd lane) alone, or Akata^−^ EBV-eGFP cells cocultured with AGS cells (4 and 5th lanes) or NU-GC-3 cells (6 and 7th lanes) directly (4 and 6th lanes) or through trans-wells (5 and 7th lanes) were purified. Four micrograms of exosomes were analyzed by Western blotting with anti-CD63 monoclonal antibody. The intensities of individual bands quantified with Fuji software are shown in the bottom.

We further investigated the role of exosomes released from epithelial cells in viral transmission by direct cocultivation for 5 days of Akata^−^ EBV-eGFP with AGS or NU-GC-3 cells in which Rab27a expression was downregulated (Figure 7A). We found that downregulation of Rab27a in both epithelial cells partly suppressed EBV transmission (Figure 7B). Taken together, all these observations indicate that exosomes released from cocultured cells contribute to some fraction of viral transmission. We also tested the effect of direct cell contact on the production of exosomes derived from cocultured cells. Exosomes isolated from the supernatants of B cells or epithelial cells alone, and from cocultured cells were subjected to western blot to determine the expression of CD63. AGS actively released exosomes compared with Akata^−^ EBV-eGFP and NU-GC-3 cells (Figure 7C). Direct cocultivation with B cells slightly increased the production of exosomes, whereas cocultivation through trans-well showed little effect on secretion of exosomes (Figure 7C).

### Role of ephrin receptor A2 on epithelial cells in cell-to-cell contact-mediated EBV transmission

**Figure 8:**
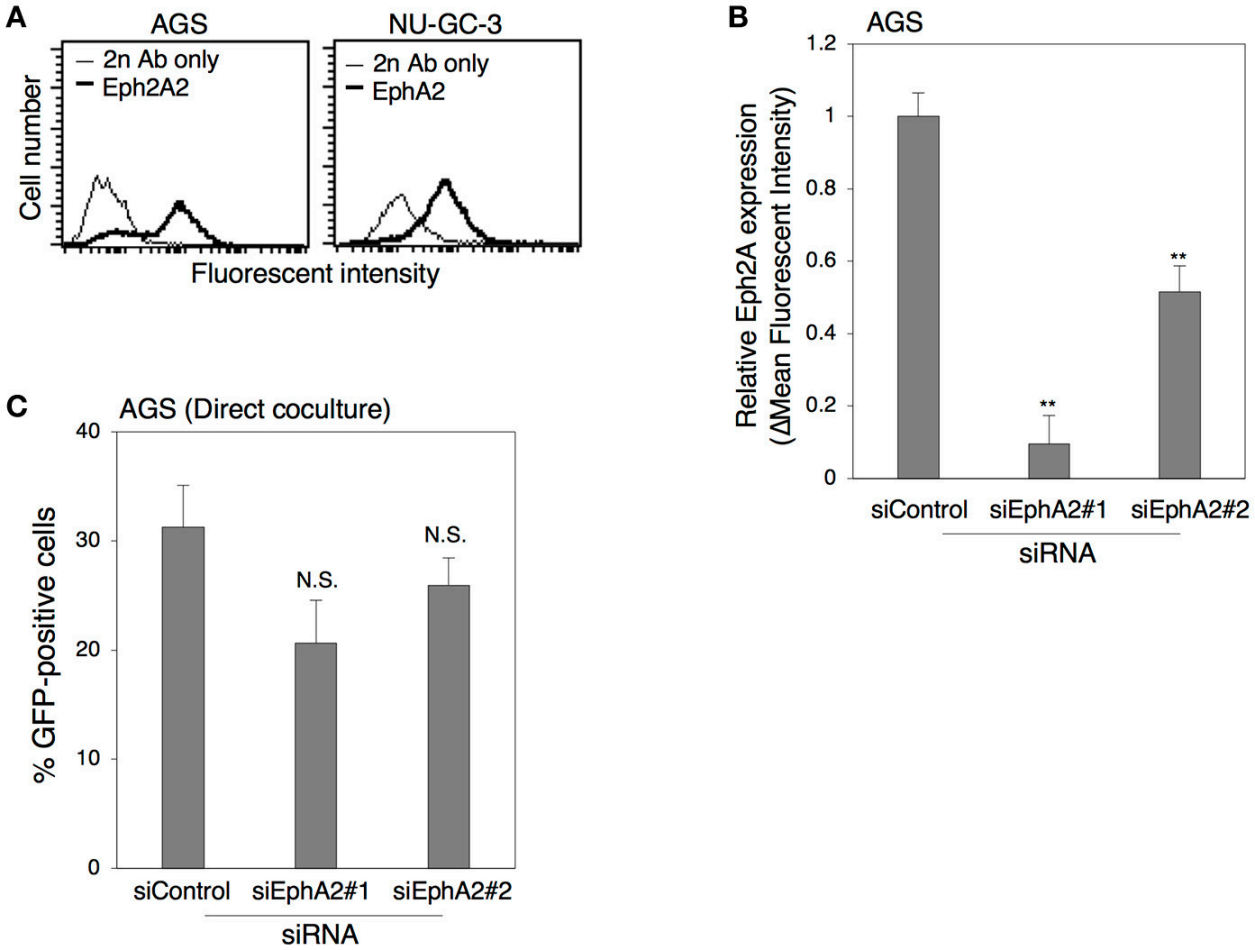
The role of Eph2A in epithelial cells in cell-to-cell contact-mediated EBV transmission. **(A)** Flow cytometric analysis for expression of Eph2A receptors in epithelial cells. Expression of Eph2A (boldface lines) in AGS (left) or in NU-GC-3 cells (right) was analyzed with flow cytometry. Representative histograms are shown. As a control, cells were incubated with the secondary antibody alone (thin lines). **(B)** Downregulation of Eph2A by siRNA in AGSC cells. Total RNA was isolated from AGS cells at 72 h-post transfection of control siRNA or two independent siRNA against Eph2A (siEph2A#1 and siEph2A #2). Downregulation of Eph2A was analyzed by flow cytometric analysis. The experiment was performed three times independently and the average and its SD are shown in each condition. ^**^P < 0.01 vs. respective control (Student's *t*-test). **(C)** Effect of downregulation of Eph2A in AGS cells on cell-to-cell contact-mediated EBV transmission. Akata^−^ EBV-eGFP cells were cocultured with AGS cells, in which Eph2A was downregulated for 5 days. The percentages of eGFP-positive, infected epithelial cells were analyzed by means of flow cytometry. The experiment was performed three times independently and the average and its SD are shown in each condition. N.S., no significant (Student's *t*-test).

Recently, the ephrin receptor A2 (EphA2) was identified as a functional receptor for EBV infection in epithelial cells (Chen et al., 2018; Zhang et al., 2018). Thus, we investigated the role of this receptor in cell-to-cell contact-mediated EBV transmission. We first examined the expression of EphA2 in AGS and NU-GC-3 cells by flow cytometric analysis. The data indicated that both AGS and NU-GC-3 cells were EphA2-positive (Figure 8A). We then knocked down the expression of EphA2 in AGS cells with two independent siRNAs (siEph2A#1 and siEph2A#2) to examine the role of EphA2 in viral transmission. We confirmed that both siRNAs, in particular siEph2A#1 significantly suppressed the expression of EphA2 by means of flow cytometry (Figure 8B). Akata^−^ EBV-eGFP were directly cocultivated for 5 days with AGS cells in which EphA2 expression was downregulated. The data indicated that downregulation of EphA2 in AGS cells exhibited little effect on viral transmission (Figure 8C), suggesting that EphA2 is dispensable for cell-to-cell contact-mediated EBV transmission.

## Discussion

In our present study, we uncovered roles for secreted factors in cell-to-cell contact-mediated EBV transmission by using an *in vitro* cocultivation-based assay. Our observations indicate that direct cocultivation of latently EBV-infected B cells and epithelial cells increases viral transmission into epithelial cells in a time-dependent manner (Figure 1), which is likely associated with the induction of the lytic cycle in cocultured B cells (Figure 2). While suppression of secretion of exosomes from EBV-positive B cells and epithelial cells partly inhibited viral transmission (Figures 6, 7), treatment with a blocking antibody for TGF-β suppressed the lytic cycle in B cells and subsequent viral transmission in a dose-dependent manner (Figure 3). Cell-contact facilitates the expression of TGF-β receptors on the surface of B cells and increases their susceptibility to TGF-β secreted from epithelial cells (Figure 5).

These findings indicate that TGF-β spontaneously released from epithelial cells fosters induction of the lytic cycle in BL cells, which leads to efficient viral transmission into epithelial cells.

Previous studies found that some BL cell lines including Akata cells are resistant to TGF-β-induced lytic cycle induction because of a possible epigenetic-mediated lack of expression of TβRII (Inman and Allday, 2000; Fukuda et al., 2006). However, we detected expression of TβRII in both EBV-positive and –negative Akata clones (Figure 4) isolated from parental Akata cells (Shimizu et al., 1994). In the process of cloning of Akata^+^ and Akata^−^ eGFP-EBV cells, we have evaluated the efficiency of viral production induced by anti-human IgG for each clone, which might allow TβRII-positive clones to be selectively isolated.

We observed that expression of cell surface TGF-β receptors is upregulated in directly cocultured B cells, which confers susceptibility to TGF-β into B cells (Figure 5). Multiple kinds of evidence show that internalization of the TGF-β receptors *via* clathrin-coated pits or lipid raft play a role in regulating TGF-β-induced signaling (Penheiter et al., 2002; Di Guglielmo et al., 2003; Chen, 2009). Recently it has been also shown that downregulation of Na^+^/K^+^ ATPase suppressed expression of TβRII in epithelial cells (La et al., 2016). We have demonstrated direct cell contact upregulates the recycling of ICAM-1 on the surface of cocultured B cells (Nanbo et al., 2016), suggesting that EBV also upregulates TGF-β receptors by modulating host membrane trafficking machinery.

We observed that the upregulation of TGF-β receptors was transient (Figure 5A). Previously, we demonstrated that direct cell contact induces transient bi-directional activation of signal transduction pathways, including the ERK pathway (Nanbo et al., 2012). The promoter region of TGF-β1 possesses putative binding sites for multiple transcription factors, such as c-fos, myc, CREB1, and c-Jun, which are known to play roles downstream of the ERK pathway, suggesting that transient upregulation of TGF-β receptors may be partly mediated by the ERK pathway.

It has been shown that the atrophic border of the gastric mucosa, where EBV-associated GC develops, frequently generates mild to moderate atrophy and attracts inflammatory cells including lymphocytes (Hirano et al., 2003). Moreover, elevated levels of TGF-β have been also reported in patients with GC and gastric precancer (Mizoi et al., 1993; Maehara et al., 1999; Ebert et al., 2000; Ma et al., 2013), which might be associated with patient survival rate (Chen et al., 2003). In the present study, we have demonstrated that TGF-β secreted from epithelial cells induces the viral lytic cycle in EBV-infected B cells (Figure 2). These observations, along with pathological and epidemiological studies, suggest that viral transmission mediated by induction of the lytic cycle in the infiltrating EBV-infected B cells cell by TGF-β derived from epithelial cells is an appropriate model for development of EBV-associated GC.

GC cells express various secretion factors including growth factors, gastrointestinal hormones, and cytokines (Mclean and El-Omar, 2014), suggesting that these secreted factors may contribute to the progression of tumors by modulating phenotypes of EBV-infected B cells in paracrine, and/or juxtacrine-dependent manner.

Several studies support a possible role for extracellular vesicles in cell-to-cell contact mediated intracellular communication such as IS-mediated antigen presentation (Mittelbrunn et al., 2011; Choudhuri et al., 2014). One study indicated that exosomes derived from T cells transfer miRNAs to APCs, which modulate gene expression in recipient cells (Mittelbrunn et al., 2011). Another study revealed that T cells shed extracellular microvesicles possessing T cell receptors toward the central cleft of IS. TCR-expressing exosomes then bound to the MHC molecules on the surface of recipient APCs, which induced activation of APCs (Choudhuri et al., 2014). Moreover, recently Lin and colleagues demonstrated that oropharyngeal epithelial cells release exosomes containing the epithelium-specific miRNA, miR-200, which are subsequently transferred to cocultured Mutu I cells and induce viral reactivation (Lin et al., 2016). Previously we demonstrated that exosomes derived from EBV-infected B cells promote ICAM-1 expression in EBV-negative epithelial cells (Nanbo et al., 2013). We also observed that various adhesion molecules including ICAM-1 contribute to the establishment of efficient cell contact-mediated EBV transmission (Nanbo et al., 2016). Based on our observations, we proposed a model in which locally secreted exosomes from EBV-positive B cells promote ICAM-1 expression, which leads to the stabilization of cell contact and subsequent viral transmission. In the present study, we found that exosomes released from EBV-infected B cells and epithelial cells partly promote EBV transmission (Figures 6, 7). It has been shown that EBV-encoded micro RNAs are expressed at higher levels in tumor biopsies than in cells grown in culture derived from them (Chen et al., 2010; Qiu et al., 2015; Yang et al., 2017), suggesting that exosomes derived from cocultured cells may function in viral transmission more efficiently than under normal physiological conditions.

Little is known about the linkage between exosomes and TGF-β signaling. A previous study, indicated that exosomes derived from chronic myeloid leukemia promote the proliferation and survival of tumor cells *in vitro* and *in vivo* by transferring TGF-β1 to the recipient cells (Raimondo et al., 2015). Another study demonstrated that the exosomes released from stromal fibroblasts derived from patients with oral cavity squamous cell carcinoma (SCC) contains TβRII and promotes TGF-β signaling in keratinocytes derived from SCC (Languino et al., 2016). We could not detect these molecules in the isolated exosomes released from cocultured cells (data not shown). Moreover, TGF-β receptors were not upregulated in B cells cocultured with AGS cells through trans-wells (Figure 5B), indicating that it is unlikely that exosomes transfer these receptors to B cells.

Previously we demonstrated that clathrin-mediated endocytosis is involved in the uptake of EBV by cocultured recipient epithelial cells (Nanbo et al., 2016), suggesting that unknown cellular receptors on epithelial cells are involved in this process. In this study, we observed that EphA2, which is recently identified as an epithelia cell receptor for entry of cell-free EBV, was dispensable for cell-to-cell contact-mediated EBV transmission (Figure 8). Further studies are needed in order to clarify the molecular mechanism underlying this process.

Taken together, our study demonstrates that EBV exploits secreted factors released from host cells for efficient viral transmission, providing new insights into the mechanism of cell-to-cell viral transmission.

## Author contributions

AN was involved in conceptualizing the study, experimental design and data analysis. AN conducted the experiments with the support of MO. MO and HY provided the materials. AN wrote the draft that was edited by MO, HY, and YO.

### Conflict of interest statement

The authors declare that the research was conducted in the absence of any commercial or financial relationships that could be construed as a potential conflict of interest.
